# Global gene expression in muscle from fasted/refed trout reveals up-regulation of genes promoting myofibre hypertrophy but not myofibre production

**DOI:** 10.1186/s12864-017-3837-9

**Published:** 2017-06-07

**Authors:** Pierre-Yves Rescan, Aurelie Le Cam, Cécile Rallière, Jérôme Montfort

**Affiliations:** INRA, UR 1037, LPGP Fish Physiology and Genomics, Campus de Beaulieu, F-35042 Rennes, France

**Keywords:** Muscle growth, Muscle hyperplasia, Muscle hypertrophy, Gene expression, Transcriptome, Teleost

## Abstract

**Background:**

Compensatory growth is a phase of rapid growth, greater than the growth rate of control animals, that occurs after a period of growth-stunting conditions. Fish show a capacity for compensatory growth after alleviation of dietary restriction, but the underlying cellular mechanisms are unknown. To learn more about the contribution of genes regulating hypertrophy (an increase in muscle fibre size) and hyperplasia (the generation of new muscle fibres) in the compensatory muscle growth response in fish, we used high-density microarray analysis to investigate the global gene expression in muscle of trout during a fasting-refeeding schedule and in muscle of control-fed trout displaying normal growth.

**Results:**

The compensatory muscle growth signature, as defined by genes up-regulated in muscles of refed trout compared with control-fed trout, showed enrichment in functional categories related to protein biosynthesis and maturation, such as RNA processing, ribonucleoprotein complex biogenesis, ribosome biogenesis, translation and protein folding. This signature was also enriched in chromatin-remodelling factors of the protein arginine N-methyl transferase family. Unexpectedly, functional categories related to cell division and DNA replication were not inferred from the molecular signature of compensatory muscle growth, and this signature contained virtually none of the genes previously reported to be up-regulated in hyperplastic growth zones of the late trout embryo myotome and to potentially be involved in production of new myofibres, notably genes encoding myogenic regulatory factors, transmembrane receptors essential for myoblast fusion or myofibrillar proteins predominant in nascent myofibres.

**Conclusion:**

Genes promoting myofibre growth, but not myofibre formation, were up-regulated in muscles of refed trout compared with continually fed trout. This suggests that a compensatory muscle growth response, resulting from the stimulation of hypertrophy but not the stimulation of hyperplasia, occurs in trout after refeeding. The generation of a large set of genes up-regulated in muscle of refed trout may yield insights into the molecular and cellular mechanisms controlling skeletal muscle mass in teleost and serve as a useful list of potential molecular markers of muscle growth in fish.

**Electronic supplementary material:**

The online version of this article (doi:10.1186/s12864-017-3837-9) contains supplementary material, which is available to authorized users.

## Background

Skeletal muscle has a number of important functions: it maintains posture, produces locomotion and is a dominant organ in energy metabolism. Skeletal muscle is a dynamic structure that forms during embryogenesis, grows afterwards and is subjected to remodeling and changes in mass under a variety of physiological conditions such as nutritional perturbations, modifications of activity or ageing. In mammals, embryonic and fetal skeletal muscle forms and grows via the proliferation, differentiation and fusion of myogenic cells, whereas postnatal muscle grows largely through remodelling of pre-existing myofibres [1]. In contrast, fish lastingly combine muscle hyperplasia (generation of new fibres) and hypertrophy (increase in fibre size) to generate indeterminate muscle growth [2, 3]. Muscle hyperplasia in fish initially occurs in a discrete layer at the surface of the myotome in late embryos or early larvae. This regionalized phase of myogenesis, termed stratified hyperplasia, results from the differentiation of myogenic progenitor cells originating from the dermomyotome-like epithelium present at the surface of the embryonic myotome [4–6]. At post-larval stages, new myofibres are formed between existing muscle fibres throughout the myotome, thus producing a typical mosaic appearance in muscle cross sections [2]. A Myog:GFP transgenic trout model has revealed that mosaic hyperplasia is prevalent in the juvenile stage, but progressively decreases as trout age, and eventually ceases at approximately 18 months post-fertilization [7]. Nevertheless, potentially recruitable muscle stem cells are present in muscles of aged trout, as shown by their ability to form myofibres de novo after muscle injury [7, 8].

Although the genetic programs determining muscle development are well characterized in vertebrates, including fish [9], little is known regarding the programs involved in muscle remodelling and muscle mass changes occurring throughout life. Among adaptive responses that remodel muscle, there is compensatory growth. Compensatory growth is a phase of rapid growth, greater than the growth rate of control animals, that follows growth depression [10]. Compensatory growth is triggered by refeeding of animals after a period of weight loss induced by a prolonged fasting period. In fish, compensatory growth has been related to an increase in feed intake (hyperphagia) [11] and efficiency of food utilization [10]. Hence, there is considerable interest in exploiting compensatory growth, through the design of feeding schedule, to optimize muscle growth [12, 13]. Using nylon macroarrays, we have previously reported a preliminary exploration of the temporal gene expression profiling of trout muscle during a fasting-refeeding schedule [14]. In the present study, using a high-density Agilent-based microarray platform for trout, and considering the gene expression of control-fed trout that displayed usual growth, we exhaustively defined genes specifically associated with the compensatory muscle growth response, and characterized the corresponding functional categories. Additionally, to further characterize the cellular mechanisms involved in the compensatory growth response, we compared the genes composing the compensatory muscle growth signature and the genes up-regulated in hyperplastic growth zones of the post-embryonic trout myotome, as they were previously identified using laser-capture microdissection combined with the same Agilent-based microarray platform [15].

## Results

### Growth characteristics in fasted, fasted-refed and control (normally) fed trout

**Fig. 1:**
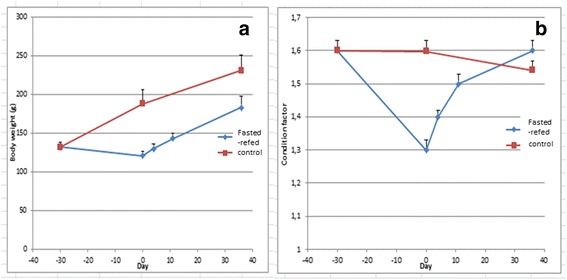
Change in body mass and condition factor over the time course of the experiment. Body weight (**a**) and condition factor (**b**) curves of trout in experimental (fasted-refed) and control (normally fed) groups. Bars indicate standard error of the mean

Changes in body weight and condition factor K, an indicator of body shape, are shown in Fig. 1. This figure shows a slight decrease in total body weight after fasting that was followed by an increase in total body weight after refeeding. Nonetheless, total body weight increase in refed animals was found to be comparable to that observed in control group from day 0 to day 36. The condition factor K decreased after nutrient deprivation and increased after refeeding to reach control level. By contrast, condition factor only moderately decreased at day 36. On the whole, these data show a resumption of growth after refeeding, but comparison of growth curves in refed and control trout did not objectify a burst of muscle growth after refeeding. To further provide experimental evidence of a compensatory growth response in muscle of refed trout, a global gene expression analysis was conducted in muscle of refed and control-fed trout.

### Gene expression profiling overview

**Fig. 2:**
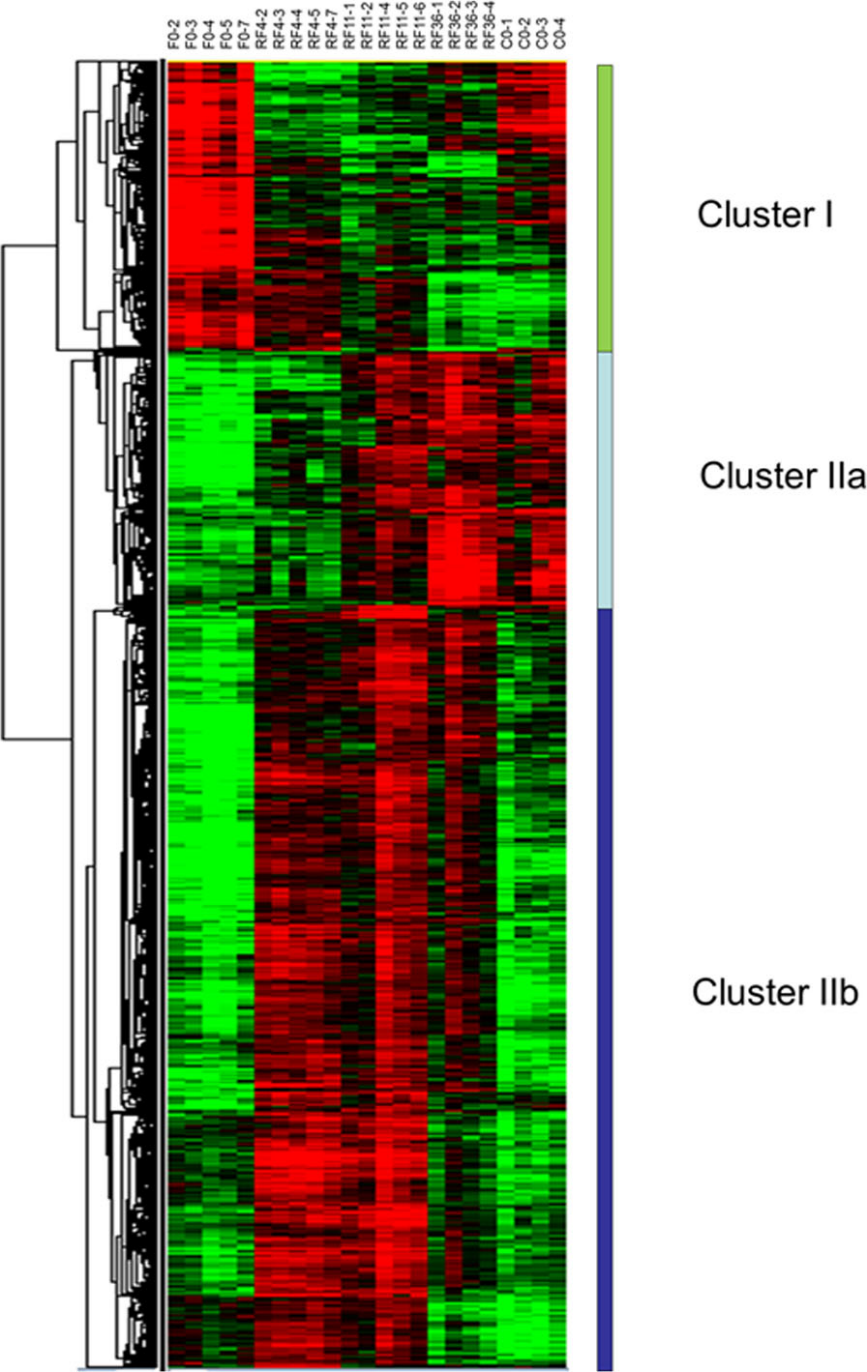
Hierarchical clustering of differentially expressed genes in muscle during a fasting-refeeding schedule and in control-fed trout displaying usual growth. Hierarchical clustering of differentially expressed genes led to the formation of three distinct clusters: I, IIa and IIb. Cluster IIb, which includes genes up-regulated in muscles of refed trout compared with control-fed trout, defines the specific molecular signature of compensatory muscle growth following refeeding. Each row represents the expression pattern of a single gene, and each column corresponds to a single sample: columns 1 to 5, muscles from fasted trout; columns 6 to 10, 11 to 15 and 16 to 19, muscles from 4-, 11- and 36-days refed trout respectively; columns 20 to 23, muscles of control-fed trout. The expression levels are represented by colored tags, with red representing the highest levels of expression and green representing the lowest levels of expression

Anova test was used to define genes whose expression levels in trout muscle were significantly different across the various feeding conditions considered (fasted for 1 month (F0), fasted for 2 month and then refed 4, 11 or 36 days (RF4, RF11 and RF36, respectively) and normally fed (control: C0). This analysis led to the identification of approximately 2300 unique differentially expressed genes. The hierarchical clustering of differentially expressed genes, shown in Fig. 2 and available through the heat map file (Additional file 1) and Java TreeView tool (https://sourceforge.net/projects/jtreeview/files/) resulted in the formation of three major clusters with distinct expression profile. Cluster I contained genes up-regulated in muscles of fasted trout and then down-regulated after refeeding. This cluster contained markers of muscle atrophy such as FBXO32/atrogin1 and trim63 (murf1), and markers of autophagy such as SQSTM1, thus indicating the activation of proteolytic systems in muscles of fasted trout. Clusters IIa and IIb both contained genes up-regulated after refeeding. Cluster IIa included genes whose expression levels after refeeding were restored to values found in control (normally fed) trout, and cluster IIb included genes whose expression levels after refeeding exceeded those in muscles of control-fed trout. Because compensatory growth refers to a faster than usual (control-fed) growth rate that is induced after refeeding of fasted animals [16], we reasoned that the compensatory muscle growth signature, consisted only of genes contained in cluster IIb. The study presented here focused on genes up-regulated after refeeding and more especially on those forming the compensatory growth signature (cluster IIb).

### Cluster IIa: Genes whose expression levels after refeeding was restored to values found in control trout

Cluster IIa was composed of 422 unique genes whose expression levels during muscle recovery progressively increased to reach those of normally fed trout. Gene ontology analysis was performed to understand the functional significance of the genes contained in cluster IIa. The DAVID analysis of 357 eligible genes specific to this cluster showed enrichment in GO terms linked to cell division (mitosis (*P* < 1.4.10^−7^), organelle fission (*P* < 2.6.10^−7^) and chromosome condensation (*P* < 3.9.10^−4^)). In addition, the genes in cluster IIa showed enrichment in GO terms related to cytoskeleton organization (*P* < 3.7.10^−6^), components of the sarcomere (*P* < 2.5.10^−5^, 12 genes including myosins, skeletal muscle actins, troponins and tropomyosins), and proteins of the extracellular matrix (*P* < 4.10^−11^, 31 genes including collagen chains, laminin subunits, periostin, CILP1, tenascin and many proteoglycans such as syndecan 2, mimecan and keratocan). Overall, cluster IIa showed enrichment in genes involved in cell division and genes encoding structural components of muscle fibres (for details, see Table 1 and Additional file 2 for list of genes that formed cluster IIa).

**Table 1:** Functional categories related to cluster IIa and cluster IIb

Cluster IIa			Cluster IIb		
Term	Count	pValue	Term	Count	*p* Value
GO Biological process			GO Biological processes		
mitosis	20	1.4.10^-7^	RNA processing RNA processing	137	5.6.10^-53^
organelle fission	20	2.6.10^-7^	ribonucleoprotein complex biogenesis	78	7.10^-49^
cell division	21	3.1.10^-6^	ribosome biogenesis	57	9.1.10^-38^
cytoskeleton organization	26	3.7.10^-6^	rRNA metabolic process	45	7.4.10^-30^
GO cellular component			translation	77	4.7.10^-27^
extracellular matrix	31	4.10^-11^	protein folding	48	7.1.10^-20^
contractile fiber part	12	2.5.10^-5^	mRNA metabolic process	70	2.8.10^-19^
condensed chromosome	11	3.9.10^-4^	tRNA metabolic process	37	1.2.10^-17^
			cellular macromolecular complex assembly	59	8.4.10^-16^
			GO cellular component		
			membrane-enclosed lumen	297	1.10^-69^
			nucleolus	140	1.1.10^-40^
			mitochondrion	138	2.10^-19^
			spliceosome	41	6.6.10^-19^
			ribosome	47	5.4.10^-15^
			small nuclear ribonucleoprotein complex	16	1.9.10^-13^

### Cluster IIb: Genes whose expression levels after refeeding exceeded those in control trout

**Fig. 3:**
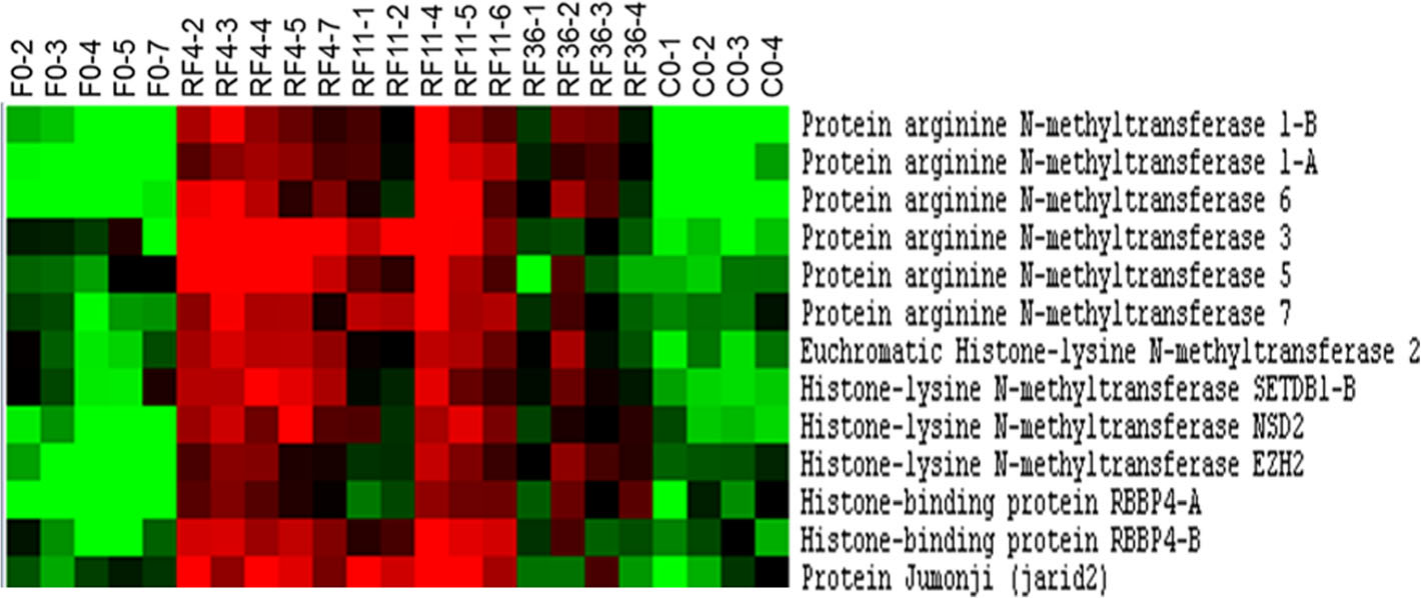
Supervised clustering of chromatin-remodeling factors present in compensatory muscle growth signature. Columns are as in Fig. 2

Cluster IIb was composed of 1161 unique genes up-regulated in muscles of refed trout compared with both fasted and control-fed trout; as such, we identified cluster IIb as the compensatory muscle growth signature. Notably, genes composing cluster IIb clearly displayed an earlier up-regulation after refeeding than genes forming cluster IIa. A DAVID analysis of 960 eligible genes showed enrichment in GO terms linked to transcription, such as RNA processing (*P* < 5.6.10^−53^), rRNA metabolic process (*P* < 7.4.10^−30^), mRNA metabolic process (*p* < 2.8.10^−19^), tRNA metabolic process (*P* < 1.2.10^−17^), and ribonucleoprotein complex biogenesis (*P* < 7.10^−49^). Other GO terms associated with cluster IIb included translation (*P* < 4.7.10^−27^), ribosome biogenesis (*P* < 9.1.10^−38^), which determines translation capacity, cellular macromolecular complex assembly (*P* < 8.4.10^−16^) and protein folding (*P* < 7.1.10^−20^). Enrichment in genes involved in mitochondria biogenesis and activity (*P* < 2.10^−19^) was also found in cluster IIb. Finally, cluster IIb included many genes encoding epigenetic transcriptional regulators. Among them were the histone-lysine N-methyltransferases EZH2, jarid2/Jumonji and RBBP4 which are all components of the PCR2 complex, the histone-lysine N-methyltransferases EHMT2, SETDB1 and WHSC1/NSD2, and many histone modifying enzymes of the protein arginine methyltransferase (PRMT) family such as Prmt1-A, Prmt1-B Prmt3; Prmt5, Prmt6 and Prmt7 (Fig. 3). Overall, the molecular signature of compensatory muscle growth showed enrichment in genes encoding histone methyltransferases, genes regulating protein biosynthesis for cell growth and genes involved in mitochondrion biogenesis for energy supply (for details, see Tables 1, 2, 3 and 4, and Additional file 3 for list of genes that that formed the major functional categories of cluster IIb).

**Table 2:** List of genes of the compensatory muscle growth signature involved in translation

ID	Gene Name	ID	Gene Name
ABCF1	ATP-binding cassette, sub-family F (GCN20), member 1	MRPL12	mitochondrial ribosomal protein L12
GFM1	G elongation factor, mitochondrial 1	MRPL17	mitochondrial ribosomal protein L17
SEPSECS	Sep (O-phosphoserine) tRNA:Sec (selenocysteine) tRNA synthase	MRPL22	mitochondrial ribosomal protein L22
TSFM	Ts translation elongation factor, mitochondrial	MRPL28	mitochondrial ribosomal protein L28
AARS	alanyl-tRNA synthetase	MRPL3	mitochondrial ribosomal protein L3
RARS	arginyl-tRNA synthetase	MRPL32	mitochondrial ribosomal protein L32
NARS	asparaginyl-tRNA synthetase	MRPL37	mitochondrial ribosomal protein L37
CARS	cysteinyl-tRNA synthetase	MRPL4	mitochondrial ribosomal protein L4
DENR	density-regulated protein	MRPL45	mitochondrial ribosomal protein L45
EEFSEC	eukaryotic elongation factor, selenocysteine-tRNA-specific	MRPL47	mitochondrial ribosomal protein L47
EEF1E1	eukaryotic translation elongation factor 1 epsilon 1	MRPL51	mitochondrial ribosomal protein L51
EIF1AX	eukaryotic translation initiation factor 1A, X-linked	MRPL52	mitochondrial ribosomal protein L52
EIF2S1	eukaryotic translation initiation factor 2, subunit 1 alpha, 35 kDa	MRPL55	mitochondrial ribosomal protein L55
EIF2B1	eukaryotic translation initiation factor 2B, subunit 1 alpha, 26 kDa	MRPS10	mitochondrial ribosomal protein S10
EIF2B3	eukaryotic translation initiation factor 2B, subunit 3 gamma, 58 kDa	MRPS12	mitochondrial ribosomal protein S12
EIF3D	eukaryotic translation initiation factor 3, subunit D	MRPS18B	mitochondrial ribosomal protein S18B
EIF3H	eukaryotic translation initiation factor 3, subunit H	MRPS25	mitochondrial ribosomal protein S25
EIF3J	eukaryotic translation initiation factor 3, subunit J	MRPS33	mitochondrial ribosomal protein S33
EIF4G1	eukaryotic translation initiation factor 4 gamma, 1	MRPS5	mitochondrial ribosomal protein S5
EIF4G2	eukaryotic translation initiation factor 4 gamma, 2	MRPS6	mitochondrial ribosomal protein S6
EIF4G3	eukaryotic translation initiation factor 4 gamma, 3	PELO	pelota homolog
EIF4E	eukaryotic translation initiation factor 4E	RSL1D1	ribosomal L1 domain containing 1
EIF4H	eukaryotic translation initiation factor 4H	RPL23	ribosomal protein L23 pseudogene 6
EIF5	eukaryotic translation initiation factor 5	RPL31	ribosomal protein L31 pseudogene 49
EIF5B	eukaryotic translation initiation factor 5B	RPL5	ribosomal protein L5 pseudogene 34
EIF6	eukaryotic translation initiation factor 6	RPL7L1	ribosomal protein L7-like 1; ribosomal protein L7 pseudogene 14
ETF1	eukaryotic translation termination factor 1	RPS9	ribosomal protein S9; ribosomal protein S9 pseudogene 4
GTF2B	general transcription factor IIB	RRBP1	ribosome binding protein 1 homolog 180 kDa (dog)
QARS	glutaminyl-tRNA synthetase	SARS2	seryl-tRNA synthetase 2, mitochondrial
GARS	glycyl-tRNA synthetase	EIF4A1	eukaryotic translation initiation factor 4A, isoform 1
HARS	histidyl-tRNA synthetase	MRPL20	similar to mitochondrial ribosomal protein L20
DTD1	D-tyrosyl-tRNA deacylase 1 homolog	TRMT6	tRNA methyltransferase 6 homolog
LARS	leucyl-tRNA synthetase	TARS	threonyl-tRNA synthetase
LGTN	ligatin	TPR	translocated promoter region (to activated MET oncogene)
KARS	lysyl-tRNA synthetase	WARS2	tryptophanyl tRNA synthetase 2, mitochondrial
MARS	methionyl-tRNA synthetase	WARS	tryptophanyl-tRNA synthetase
MRP63	mitochondrial ribosomal protein 63	YARS2	tyrosyl-tRNA synthetase 2, mitochondrial
MRPL10	mitochondrial ribosomal protein L10	VARS	valyl-tRNA synthetase
MRPL11	mitochondrial ribosomal protein L11

**Table 3:** List of genes of the compensatory muscle growth signature involved in ribosome biogenesis

ID	Gene Name	ID	Gene Name
C1D	C1D nuclear receptor co-repressor;	AATF	apoptosis antagonizing transcription factor
DDX51	DEAD (Asp-Glu-Ala-Asp) box polypeptide 51	BRIX1	brix domain containing 2
DDX56	DEAD (Asp-Glu-Ala-Asp) box polypeptide 56	RPF1	brix domain containing 5
DIMT1L	DIM1 dimethyladenosine transferase 1-like	BYSL	bystin-like
EBNA1BP2	EBNA1 binding protein 2	EIF6	eukaryotic translation initiation factor 6
FCF1	FCF1 small subunit (SSU) processome component homolog	EXOSC10	exosome component 10
FTSJ3	FtsJ homolog 3	EXOSC2	exosome component 2
HEATR1	HEAT repeat containing 1	EXOSC3	exosome component 3
IMP3	IMP3, U3 small nucleolar ribonucleoprotein, homolog	EXOSC4	exosome component 4
IMP4	IMP4, U3 small nucleolar ribonucleoprotein, homolog	EXOSC7	exosome component 7
KRR1	KRR1, small subunit (SSU) processome component, homolog)	EXOSC8	exosome component 8
MPHOSPH10	M-phase phosphoprotein 10 (U3 small nucleolar ribonucleoprotein	FBL	fibrillarin
MINA	MYC induced nuclear antigen	GNL3L	guanine nucleotide binding protein-like 3 (nucleolar)-like
NHP2L1	NHP2 non-histone chromosome protein 2-like 1	MRTO4	mRNA turnover 4 homolog
NOP14	NOP14 nucleolar protein homolog	NIP7	nuclear import 7 homolog
NOP2	NOP2 nucleolar protein homolog	NPM1	nucleophosmin 1 (nucleolar phosphoprotein B23, numatrin)
NOP56	NOP56 ribonucleoprotein homolog	PES1	pescadillo homolog 1, containing BRCT domain
NOP58	NOP58 ribonucleoprotein homolog	POP4	processing of precursor 4, ribonuclease P/MRP subunit
SDAD1	SDA1 domain containing 1	PDCD11	programmed cell death 11
TSR2	TSR2, 20S rRNA accumulation, homolog	PA2G4	proliferation-associated 2G4, 38 kDa;
UTP11L	UTP11-like, U3 small nucleolar ribonucleoprotein	PIN4	protein (peptidylprolyl cis/trans isomerase) NIMA-interacting, 4 (parvulin)
UTP14A	UTP14, U3 small nucleolar ribonucleoprotein, homolog A	RRP1	ribosomal RNA processing 1 homolog
UTP15	UTP15, U3 small nucleolar ribonucleoprotein, homolog	RRP1B	ribosomal RNA processing 1 homolog B
UTP18	UTP18, small subunit (SSU) processome component, homolog	RRP8	ribosomal RNA processing 8, methyltransferase, homolog
UTP23	UTP23, small subunit (SSU) processome component, homolog	RRP9	ribosomal RNA processing 9, small subunit (SSU) processome component
UTP6	UTP6, small subunit (SSU) processome component, homolog	RPL5	ribosomal protein L5
WDR12	WD repeat domain 12	SURF6	surfeit 6
WDR36	WD repeat domain 36	TBL3	transducin (beta)-like 3
DCAF13	WD repeats and SOF1 domain containing

**Table 4:** List of genes of the compensatory muscle growth signature involved in protein folding

ID	Gene Name	ID	Gene Name
Sel15	15 kDa selenoprotein	HSPD1	heat shock 60 kDa protein 1 (chaperonin)
AHSA1	AHA1, activator of heat shock 90 kDa protein ATPase homolog 1	HSPA4L	heat shock 70 kDa protein 4-like
BAG4	BCL2-associated athanogene 4	HSPA8	heat shock 70 kDa protein 8
DNAJA2	DnaJ (Hsp40) homolog, subfamily A, member 2	HSP90AA1	heat shock protein 90 kDa alpha (cytosolic), class A member 1
DNAJA4	DnaJ (Hsp40) homolog, subfamily A, member 4	TCP1	hypothetical gene supported by BC000665; t-complex 1
DNAJB9	DnaJ (Hsp40) homolog, subfamily B, member 9	LMAN1	lectin, mannose-binding, 1
DNAJC2	DnaJ (Hsp40) homolog, subfamily C, member 2	MPDU1	mannose-P-dolichol utilization defect 1
DNAJC21	DnaJ (Hsp40) homolog, subfamily C, member 21	PIN1	peptidylprolyl cis/trans isomerase, NIMA-interacting 1
FKBP11	FK506 binding protein 11, 19 kDa	PPIL1	peptidylprolyl isomerase (cyclophilin)-like 1
FKBP2	FK506 binding protein 2, 13 kDa	PPIB	peptidylprolyl isomerase B (cyclophilin B)
FKBP5	FK506 binding protein 5	PPID	peptidylprolyl isomerase D
FKBP7	FK506 binding protein 7	PPIE	peptidylprolyl isomerase E (cyclophilin E)
RANBP2	RAN binding protein 2	PPIG	peptidylprolyl isomerase G (cyclophilin G)
RUVBL2	RuvB-like 2 (*E. coli*)	PPIH	peptidylprolyl isomerase H (cyclophilin H)
CANX	calnexin	PPWD1	peptidylprolyl isomerase domain and WD repeat containing 1
CALR	calreticulin	PFDN4	prefoldin subunit 4
CCT2	chaperonin containing TCP1, subunit 2 (beta)	PFDN5	prefoldin subunit 5
CCT3	chaperonin containing TCP1, subunit 3 (gamma)	PFDN6	prefoldin subunit 6
CCT4	chaperonin containing TCP1, subunit 4 (delta)	PIN4	protein (peptidylprolyl cis/trans isomerase) NIMA-interacting, 4 (parvulin)
CCT5	chaperonin containing TCP1, subunit 5 (epsilon)	PDIA5	protein disulfide isomerase family A, member 5
CCT6A	chaperonin containing TCP1, subunit 6A (zeta 1)	PPIA	similar to TRIMCyp; peptidylprolyl isomerase A (cyclophilin A)
CCT6B	chaperonin containing TCP1, subunit 6B (zeta 2)	DNAJC19	DnaJ (Hsp40) homolog, subfamily C, member 19
HSPE1	heat shock 10 kDa protein 1 (chaperonin 10)	TOR1A	torsin family 1, member A (torsin A)
		VBP1	von Hippel-Lindau binding protein 1

### The compensatory muscle growth signature does not include genes involved in the formation of new myofibres

**Fig. 4:**
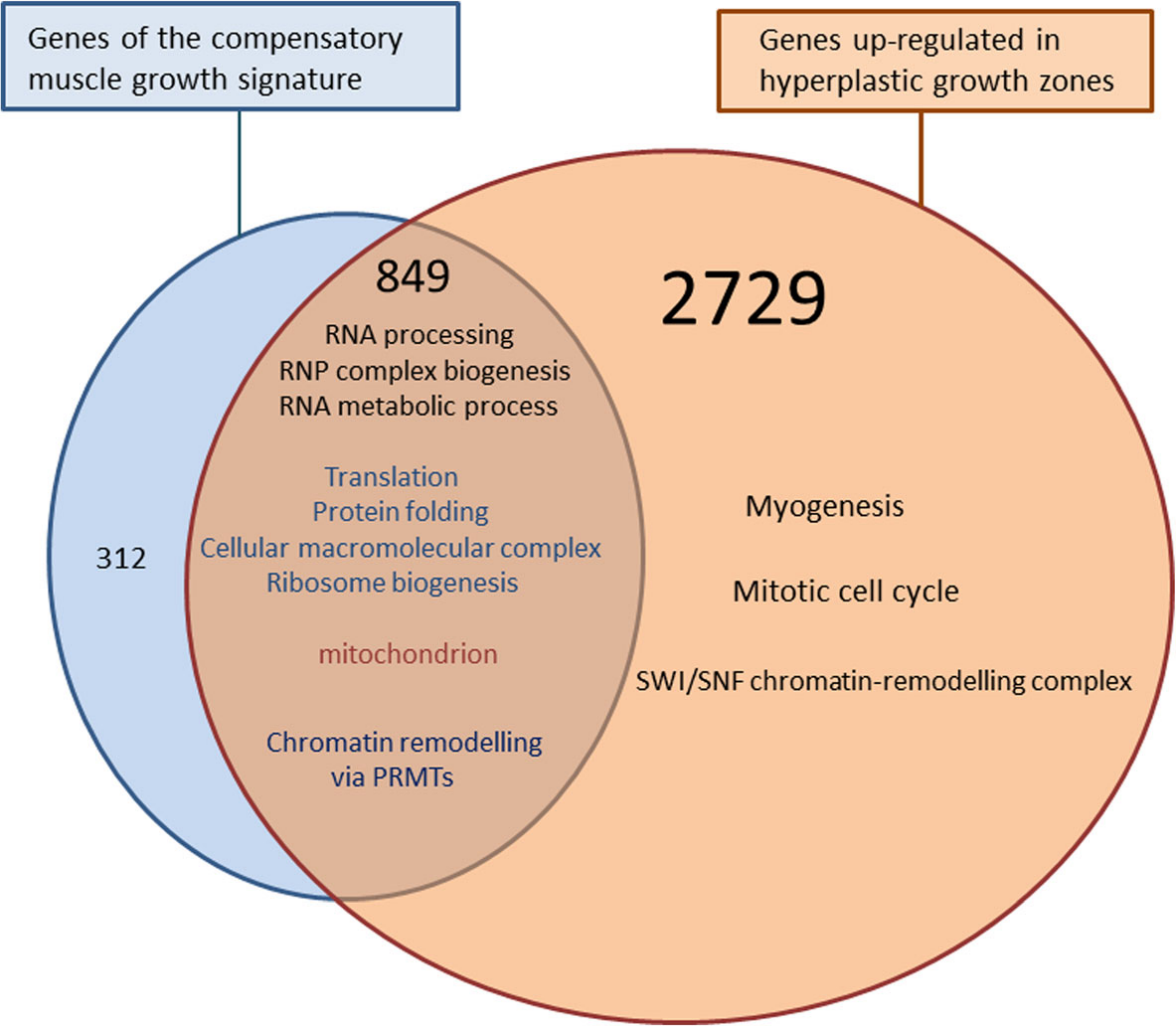
The compensatory muscle growth response involves only a subpart of the molecular signature of the hyperplastic growth zone. Venn diagram representing the distribution of genes of the compensatory muscle growth signature and genes up-regulated in the superficial hyperplastic growth zone of the late trout embryo. Functional categories inferred from genes common to the compensatory muscle growth and the hyperplastic growth zone signatures are detailed and major functional categories specific to hyperplastic growth zones are mentioned. The 312 genes specific of the compensatory muscle growth response were mostly related to translation, protein folding, RNA processing and ribosome biogenesis

To characterize the genetic mechanisms regulating the formation of new myofibres in fish we previously examined the transcriptome of the superficial hyperplastic growth zones of the late trout embryo myotome by using laser capture microdissection and microarray analysis [15]. In this study, to further characterize the cellular mechanisms involved in the compensatory growth response, we compared its molecular signature with that of the superficial hyperplastic growth zones. A Venn diagram showed that most of the genes (849 out of 1161) of the compensatory muscle growth signature were included in the list of genes up-regulated in superficial hyperplastic growth zones (Fig. 4). As a result, GO categories inferred from genes common to both situations were mostly those inferred from the compensatory growth signature, and were related to protein biosynthesis and maturation such as RNA processing, ribonucleoprotein complex biogenesis, ribosome biogenesis, translation and protein folding. Additionally, several epigenetic factors of the protein arginine N-methyl transferase (PRMT) family (i.e., Prmt1-A, Prmt-B Prmt3; Prmt5, Prmt6 and Prmt7), as well as several histone lysine N-methyltransferases (EZH2, EHMT2, WHSC1/NSD2 and SETDB1), were found in both situations. In contrast, none of the genes up-regulated in hyperplastic growth zones and that encode SWI/SNF chromatin-remodelling enzymes (i.e., Smarcd1, Smarce1, Smarcb1A, smarca5, smarcad1, Smarcab1, Smarcc1 and Smarca4/BRG1) appeared in the compensatory growth signature. This showed that only a subset of the chromatin-remodelling factors up-regulated in hyperplastic growth zones, was present in the compensatory muscle growth signature. Unexpectedly, GO terms related to DNA replication and cell cycle and that were inferred from genes up-regulated in hyperplastic growth zones were not found in the compensatory muscle growth signature. Additionally, transcriptional regulators, immunoglobulin domain-containing transmembrane proteins and secreted/signalling molecules known or predicted to be important in the differentiation of myogenic cells or their fusion into new myofibres were virtually absent from the compensatory growth signature. For example, neither canonical myogenic transcriptional regulators (Pax3, Pax7 and MRFs such as MyoD1a, MyoD1b and MyoD1c, myf5, myogenin and mrf4), nor genes encoding transcriptional regulators known to modulate MRF activity, such as Tsh3, ARX, meis1 and pbx1, were found to be associated with compensatory growth, whereas they were up-regulated in hyperplastic growth zones. Additionally, among the >30 genes found to be specific to the superficial growth areas and to encode homeobox-containing transcriptional regulators or members of the Hairy and enhancer of split family, none were included within the compensatory growth signature. Of note, Hairy and enhancer of split 6 and myogenin were found to exhibit increased expression after refeeding, but their transcription levels were never above those found in control animals. With the notable exception of M-cadherin, hyperplasia-correlated genes encoding promyogenic membrane receptors such as N-cadherin, Brother of CDO, NCAM and protogenin [17], or the essential fusion effectors Kin of Irre like-3, and Jamb [18, 19] were also absent from the compensatory muscle growth signature. Similarly, with the exception of stromal cell-derived factor 2-like protein 1 and Hepatoma-derived growth factor-related protein, virtually all the hyperplasia-correlated genes encoding secreted factors were absent from the compensatory signature. Finally, because the same Agilent-based microarray was used for gene expression profiling in both experiments, we reliably determined that among the >30 distinct genes encoding myofibrillar proteins (e.g., troponins, myosin chains, myosin binding proteins, tropomyosins, alpha actins, myozenins) that were up-regulated in laser-captured hyperplastic growth zones, only one (tropomyosin α4-chain) was found in the compensatory muscle growth signature (cluster IIb), and none were found among the numerous myofibrillar protein genes found in cluster IIa. Together, these data showed that genes common to superficial hyperplastic growth zone and to the compensatory muscle growth signature were related to protein biosynthesis for myofibre growth. In contrast, hyperplasia-correlated genes involved in myofibre production or encoding myofibrillar proteins specific to nascent myofibres were virtually excluded from the compensatory growth signature.

## Discussion

Until now, very little was known regarding the muscle growth pattern during a fasting-refeeding sequence, essentially because cellularity analysis is difficult to interpret in this case. Indeed, small muscle fibres result either from the production of new fibres (muscle hyperplasia) or from the shrinking of pre-existing fibres (muscle fibre atrophy). To gain insight into the relative contribution of hypertrophy and hyperplasia in the compensatory muscle growth response, we performed transcriptomic analysis in muscles of fasted, refed and control-fed trout and specifically investigate the expression of genes potentially involved in these two processes. Although our experimental protocol was designed to exacerbate compensatory growth (i.e., long fasting period and large excess of food during refeeding), growth curve in refed trout did not show the idealized pattern of growth compensation as presented in reviews in the field [10, 20]. Methodological problems in studies on compensatory growth have however been raised when considering growth trajectories [10]. And it is noteworthy that an absence of clear compensatory growth trajectory has been previously reported in fasted/refed trout [21, 22] and tilapia [23]. Changes in body shape (condition factor) was more suggestive of a compensatory response in our study, but food present de novo in the digestive tract of refed animals likely induced biased weights and condition factors. On the other hand, one cannot formally exclude that the experimental period chosen in our study was too limited to allow the full expression of the compensatory growth potential. Anyway, that a specific compensatory muscle growth response occurred in trout after refeeding was suggested by the identification in refed animals of a muscle molecular signature enriched in genes promoting muscle growth. Gene expression profiling overview showed that genes up-regulated after refeeding fell into two distinct categories: those whose expression level was restored to the values found in control-fed trout displaying normal growth and those whose expression level exceeded that found in control trout. Given that compensatory growth refers to a faster than usual growth rate [10], we reasoned that only genes in the second category formed the compensatory muscle growth signature, whereas genes of the first category signaled the restoration of cellular mechanisms involved in normal growth. The GO terms associated with genes whose expression was restored only after refeeding included cell division, organelle fission and condensed chromosome, thus suggesting that cell proliferation decreased in muscle during fasting but resumed after refeeding. Whether the resumption of cell proliferation after refeeding targets myogenic cells remains to be determined. However, a stimulation of myogenic cell proliferation has been reported in the Antartic fish *Notothenia coriiceps* after feeding [24]. In line with results from previous studies on muscle transcriptome dynamics during fasting-induced recovery growth [14, 25], we observed, in refed trout, the up-regulation of genes encoding structural components such as sarcomeric proteins and matricial compounds. However, we found that this up-regulation was limited to a restoration of the expression level found during normal growth. Therefore, as in the case of cell cycle regulators, matricial compounds and myofibrillar proteins were excluded from the compensatory growth signature. In sharp contrast, a very large number of genes stimulating ribosome biogenesis or enhancing translational efficiency were up-regulated in compensatory muscle growth compared to normal growth. This finding strongly suggested that the compensatory growth response was associated with an accretion of the protein mass necessary for muscle fibre hypertrophy. In agreement with this finding, a correlation has been recently established between ribosome biogenesis and the magnitude of fibre hypertrophy in overloaded mouse skeletal muscle [26], and emerging evidence supports the view that ribosome biogenesis is a crucial mechanism used by skeletal muscle to regulate protein synthesis and control muscle mass [27]. Our study further showed that the capacity to convert nascent polypeptides into functional three-dimensional structures also increased, as shown by the up-regulation of a large number of genes involved in this process, notably HSP90 and HSP70. HSP90 is required for myofibril assembly in developing zebrafish embryos [28], and its expression has been shown to increase during muscle hypertrophy resulting from functional overload in rats and mice [29]. Recently, Hsp70-null mice have been reported to display a deficit in muscle fibre size [30]. Additionally, in agreement with protein synthesis and cellular growth, which require adjustments in mitochondrial ATP production, many genes involved in mitochondrial biogenesis were found in the compensatory muscle growth signature. This finding is in line with previous morphometric analyses showing an increase in mitochondrial volume density during compensatory muscle hypertrophy produced by tenotomy of the tibialis anterior muscles of rats [31]. Palstra et al. have reported in zebrafish that muscle fibre hypertrophy promoted by swimming-induced exercise is associated with an activation of the myogenic program [32]. In contrast, we found here that the up-regulation of genes promoting myofibre hypertrophy was not associated with an activation of genes involved in myofibre production. In particular, myogenic regulatory factors of the MyoD family (MyoD, myogenin, myf5 and mrf4), which constitute a cross-regulatory transcriptional network at the core of myogenesis [33], were not found in the compensatory growth response. In line with this observation, Johansen and Overturf have reported only very few changes in MyoD1b and myf5 expression in trout muscle during refeeding following starvation [34]. In addition, the two Ig-domain transmembrane proteins Kin of Irre like3 and jamb which are essential for myocyte fusion in zebrafish embryo [18, 19], and the genes encoding contractile proteins prevalent in hyperplastic growth zones were absent from the compensatory muscle growth signature. Finally, the compensatory muscle growth signature was found to share only a subset of the chromatin-remodelling factors evidenced in hyperplastic growth zones. In particular, the SWI/SNF chromatin-remodelling enzymes up-regulated in hyperplastic growth zones were totally absent from this signature. This finding and the demonstration that Brg1/smarca4-SWI/SNF complexes are key epigenetic determinants of skeletal muscle differentiation [35] strengthen the notion that the steeper growth rate associated with compensatory growth does not involve an increase in myogenic cell differentiation. Contrasting to SWI/SNF components, many histone N-methyl transferases were both up-regulated in hyperplastic growth zones and present in the compensatory growth signature. Among them were members of the protein arginine M-methyltransferases family. Nine distinct protein arginine M-methyltransferases have been identified in mammals [36] and only Prmt4/carm1 and Prmt5 have been reported to exert myogenic activity [37, 38]. Both Prmt4/carm1 and Prmt5 were up-regulated in hyperplastic growth zones but only Prmt5 was found in the compensatory muscle growth signature. How chromatin-remodelling factors regulate the expression of genes involved in the compensatory muscle growth response will be an interesting topic for futures studies. Together, all these data strongly suggest that the steeper growth rate associated with compensatory growth results from the stimulation of hypertrophy rather than the stimulation of hyperplasia. Of note, the compensatory muscle growth signature strongly differs from the transcriptome associated with regenerating muscle [8]; the latter shows redeployment of the transcriptional program involved in myogenesis or myofibrillogenesis of nascent myotubes [8]. This result indicates that distinct genetic pathways may be activated to drive distinct aspects of the growth and remodeling of adult fish muscle.

## Conclusion

Using an Agilent-based microarray platform, we identified genes composing the molecular signature of muscle growth in fasted/refed trout and found that they were relevant to functional categories mostly involved in protein synthesis and accretion. In contrast, genes up-regulated in hyperplastic growth zones of trout muscle and that are known or predicted to be important in the formation of new myofibres were virtually absent from this signature. These findings suggest that a burst of muscle growth occurs in refed trout which is mediated by stimulation of myofibre hypertrophy. The generation of a large set of genes composing the compensatory muscle growth signature expands our understanding of the molecular and cellular mechanisms controlling skeletal muscle mass, and provide a useful list of potential molecular markers of muscle growth in fish.

## Methods

### Animals and experimental design

A spring strain of l-year-old rainbow trout (*Oncorhynchus mykiss*) was used.Trout were continually fed to satiation until the beginning of the experiment, at which time two groups of trout weighing approximately 130 g were constituted and reared in two separate tanks containing 2000 l of freshwater each. The experimental group was deprived of food for 30 days and refed for 36 days at a rate three times higher than satiation ration to exacerbate growth resumption. Trout of the control group were continually fed to satiation until the end of the experiment. Fasted fish and refed fish were taken from the experimental group at 0 day, 4 days, 11 days and 36 days following refeeding (termed F0, RF4, RF11 and RF36, respectively) with 10 fish sampled at each time point. Control-fed trout were taken 30 days (termed C0) and 66 days after the beginning of the experiment with 10 fish sampled at each time point. Fish were exposed to natural photoperiod and fed with a commercial diet (BioMar, Nersac, France). The fish were rapidly anaesthetized with phenoxy-ethanol (Aquaveto, 4 ml per 10 l of fresh water) before total body weighing, length measurement and muscle sampling (10 fish at each time point). The condition factor (an indicator of the body shape) was calculated as follows: K = body weightx100/body length 3 (the body length did not include the caudal fin length). Growth trajectories and condition factors were calculated from the 10 samples taken at each time point.

### RNA extraction, labelled cRNA preparation, hybridisation

RNA samples of five distinct trout per feeding conditions (F0, RF4, RF11, RF36 and C0) were used for microarray experiments. A transverse slice of fast muscle situated just beneath the dorsal fin was taken for RNA extraction using TRIzol (Invitrogen, Carlsbad, CA, USA) reagent following the manufacturer’s instructions. Cy3-labelled cRNA generation and hybridisation were performed as previously described [15]. A control sample that did not give signal on microarray was discarded from the gene expression analysis.

### Microarray slides

An Agilent-based microarray platform with 8 × 60 K probes per slide was used. This platform (GEO platform record: GPL15840) that was based on a rainbow trout resource designed by Yao and colleagues [39] was enriched with oligonucleotides designed from recent trout NGS data (http://ngspipelines-sigenae.toulouse.inra.fr:9064/). Microarray data sets have been submitted to the GEO-NCBI with the accession number: GSE91048.

### Data acquisition and analysis

After hybridisation, the slides were rinsed and scanned at a 3 μm resolution with the Agilent DNA Microarray Scanner. Fluorescence intensity was calculated using the standard procedures found in the Agilent Feature Extraction (FE) software version 10.7.3.1. The arrays were normalised (scale normalisation) and log-transformed using GeneSpring software (version 12.6.1). An ANOVA analysis (Benjamini-Hochberg (BH) corrected pval < 0.05) was used to determine the genes that were differentially expressed between feeding conditions. Gene expression profiles were used to classify genes and biological samples using a hierarchical clustering method using CLUSTER software and the results were visualised with TREEVIEW [40]. GO enrichment analysis was performed using Database for annotation, Visualisation and integrated Discovery (DAVID 6.7) software tools [41, 42].

## Additional files

Additional file 1: Heat map file for Java treeview visualisation of hierarchical clustering of differentially expressed genes in muscle during a fasting-refeeding schedule and in control-fed trout displaying usual growth (CDT 1214 kb)

Additional file 2: Major functional categories of cluster IIa and lists of genes that formed them (XLSX 16 kb)

Additional file 3: Major functional categories of cluster IIb and lists of genes that formed them (XLSX 59 kb)

## Abbreviations

DAVID: Database for Annotation, Visualization and Integrated Discovery
DNA: DesoxyriboNucleic acid
NCBI: National Center for Biotechnology Information
RNA: RiboNucleic acid GO: Gene ontology
